# Gender differences in horizontal strabismus: Systematic review and meta-analysis shows no difference in prevalence, but gender bias towards females in the clinic

**DOI:** 10.7189/jogh.13.04085

**Published:** 2023-09-01

**Authors:** Sydney C Laughton, Molly M Hagen, Wei Yang, Christopher S von Bartheld

**Affiliations:** 1 Center of Biomedical Research Excellence in Cell Biology, University of Nevada, Reno School of Medicine, Reno, Nevada, USA; 2 School of Public Health, University of Nevada, Reno, Nevada, USA; 3 Department of Physiology and Cell Biology, University of Nevada, Reno School of Medicine, Reno, Nevada, USA

## Abstract

**Background:**

Strabismus is a misalignment of the visual axis that affects 2-3% of the population and can lead to loss of binocular vision. It is currently controversial whether there is a gender difference in the most common form of visual misalignment: horizontal strabismus. Some studies claimed that more females than males have an outward deviation (exotropia), while others concluded that there is no significant gender difference. No previous work has systematically explored gender differences in horizontal strabismus or has compared the results of population-based studies with those of clinic-based studies.

**Methods:**

We conducted a systematic review and meta-analysis of studies reporting the prevalence of horizontal strabismus. We included 73 population-based studies and compared their disclosed gender population with that in 141 comparable clinical-based studies. We analysed the data according to gender, strabismus type (esotropia, exotropia), and geographic region/ethnicity.

**Results:**

Summary statistics showed a nearly identical prevalence of horizontal strabismus (2.558% for males, 2.582% for females), esotropia (1.386% males vs. 1.377% females), and of exotropia (1.035% males vs. 1.043% females). Meta-analysis results showed that these differences between males and females were not statistically significant (odds ratio (OR) = 1.01; 95% confidence interval (CI) = 0.97-1.10), but that females were significantly more frequent (by 7.50%) in clinic-based studies than males, with 5.00% more females for esotropia, and 12.20% more females for exotropia when adjusted for the population’s sex ratio. The extent of the female gender bias differed between geographic regions/societies, with Asians having the lowest bias towards females and Latin American countries having the strongest bias.

**Conclusions:**

Males and females have the same prevalence of horizontal strabismus, including exotropia. Females with strabismus seek health care or are brought to clinics significantly more often than males. This is an example of gender bias in health care in favour of females rather than males, apparently because parents – erroneously fearing only cosmetic consequences – are more concerned about strabismus in their daughters than their sons. Societal attitudes towards females, as well as economic factors (insurance status), appear to be relevant factors that determine the magnitude of the gender bias in horizontal strabismus.

Strabismus is a misalignment of the visual axis that affects about 2-3% of the population. Several studies over the last 80 years found a gender difference in horizontal strabismus, with many reporting that the percentage of females with exotropia exceeded that of males [1-13]. However, all but one [10] were clinical- rather than population-based and therefore did not provide information about the prevalence of strabismus, but rather which gender more frequently seeks medical attention. It is also possible that reported gender differences are spurious, produced by small cohort sizes. Indeed, the population-based studies with the largest cohorts reported no significant difference in the prevalence of horizontal strabismus between males and females [14-16]. Furthermore, several population and clinic-based studies reported more males than females for overall horizontal strabismus [17] or for exotropia [18], or detected no gender difference [14-16,19-22]. Since the question of gender differences in horizontal strabismus has never been systematically explored or conclusively resolved, it is currently not clear whether gender differences exist, overall, or only for certain types of strabismus (exotropia), or, if they do exist, whether they are restricted to certain populations. Importantly, no previous study has explored whether there is a gender difference between the prevalence of horizontal strabismus (according to population-based studies) and the frequency with which patients seek medical attention (according to clinic-based studies). It is conceivable that gender differences in the diagnosis and treatment of horizontal strabismus may reflect cultural beliefs and norms that differ between populations [9,11,23-25]. To resolve these questions, we conducted a systematic review of population- and clinic-based studies on horizontal strabismus. Our meta-analysis on the gender distribution in both types of studies (population-based and clinic-based) shows that there is no gender difference in horizontal strabismus prevalence, yet there is a significant gender bias towards females in the clinic. We further identified ethnicity or geographic region as a relevant factor that impacts female gender bias in the clinic, and we discuss the likely underlying reasons for this example of bias towards females in health care.

## METHODS

### Systematic review

**Figure 1:**
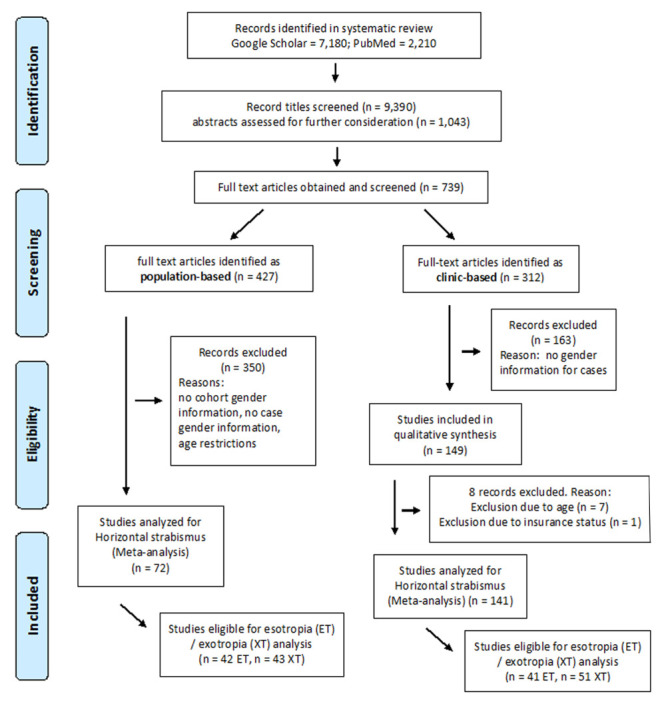
Flowchart illustrating the literature search, systematic review and meta-analysis according to the PRISMA guidelines. PubMed and Google Scholar were searched systematically. The literature search was last updated on 8 September 2022.

We searched PubMed and Google Scholar (last updated on 8 September 2022) for studies quantifying the prevalence and frequencies of horizontal strabismus and also screened the relevant citations of the retrieved studies. We used keywords such as strabismus, prevalence, esotropia, and exotropia without setting restrictions on the type of publication, year of publication, or language. We found and considered studies in 22 different languages; for languages with which we were not familiar, we asked colleagues to provide translations. A total of 9390 titles were screened; after removal of duplicates, 1043 abstracts were examined, and after reviewing the full text of 739 of these records, they were divided into population-based studies (n = 427) and clinic-based studies (n = 312). The 427 population-based studies reporting on the prevalence of horizontal strabismus were screened for gender information, specifically for the disclosure of gender in cohorts and the disclosure of gender in strabismus cases. Likewise, all 312 clinic-based studies of horizontal strabismus were screened for gender information (**Figure 1**). Both types of studies were further examined for the presence of more detailed information regarding horizontal strabismus types (esotropia and exotropia), but this differentiation was not a required inclusion criterium. We followed the PRISMA guidelines for systematic searches [26], as illustrated in the flowchart (**Figure 1**). All studies with gender information that were identified as either population-based or clinic-based are listed alphabetically by author in Table S1 and S2 in the **Online Supplementary Document**.

### Data extraction

We considered studies “population-based” only when populations or representative samples were screened for visual misalignment, but not when the source of data was from clinics or medical records, in which case the study was considered for the category “clinic-based.” Any disagreements about data extraction and categorisation were discussed and resolved among the team of authors. Inclusion criteria for population-based studies were that quantitative information about the male-female distribution was provided for a representative cohort, as well as quantitative information about the male-female distribution for cases of horizontal strabismus. We included clinical-based studies that provided quantitative information about the male-female distribution of patients with horizontal strabismus and excluded all that reported data only on males or only on females. We use the terms “male/female” rather than “men/women” or “boys/girls” because “male/female” is age-neutral, and many studies do not distinguish ages within cohorts.

### Age

Although most strabismus develops in early childhood and most studies target children and adolescents, we initially compiled all studies regardless of age range. Only one of the eligible population-based studies focused on older age, while seven of the 149 eligible clinic-based studies examined exclusively adults over the age of 50 years. To make the two types of studies directly comparable, eight older-age studies (one population-based study, and seven clinic-based studies) were excluded from the meta-analyses.

### Sex ratio

The sex ratio differs significantly between societies and countries [27]. Knowing the sex ratio in a country is important because, with a larger fraction of males than females, males would be expected more often in clinics than females, given that the prevalence of horizontal strabismus is the same between males and females. In the age group of children and young adults, all countries have more males than females, but the amount of excess males differs between countries [27,28]. To adjust for this gender difference, we estimated the true gender difference for each study by calculating the expected number of females based on the sex ratio for that country, and compared that number with the observed number of females relative to males; any excess or deficit was then entered as the adjusted value for the females relative to that of males in that study. The information about sex ratios for the different countries was obtained from a 2021 United Nations data bank for the age group zero to 14 years [28].

### Statistical analysis

All meta-analyses were carried out in the software R, version 3.6.1 (R Foundation for Statistical Computing, Vienna, Austria). To calculate estimates of pooled prevalence, odds ratios (OR), and 95% confidence intervals (CI), we used the R-meta package, version 4.9-5. For the population-based data, we used the metabin function with the adjusted number of females and the number of males with any horizontal strabismus/exotropia/esotropia as the “control” and “intervention” event count data, and the total number of males and females in each study as the sample size for the “control” and “intervention” groups [29]. This allowed us to calculate pooled odds ratios with 95% CI for the odds of each ocular condition (all horizontal strabismus, exotropia, and esotropia) for females relative to males in the population-based studies. For the clinic-based data, because all individuals presented with a strabismus condition, we used a different methodology and estimated the proportion of males vs. the proportion of females using the metaprop function and the adjusted female count data [29]. For both the population-based and clinic-based data, we used random effects models with the inverse variance method for pooling and subgroup tests to evaluate regional differences in trends [30]. For both data sets, analysis of the heterogeneity across studies was done using the Maximum-likelihood estimator, Higgins’ *I^2^* and Cochran’s *Q* method [30,31]. Publication bias was assessed by visual inspection and a rank-correlation test of funnel plots as shown in Figure S3, panels A and B, in the **Online Supplementary Document**. In all cases, significance was assessed at *P*- value = 0.05 with a 95% CI.

### Region/ethnicity

Subgroup comparisons examined the effect of ethnicity or geographical region. To detect potential differences between ethnicities, we grouped studies by geography as follows: Western countries (mostly people of European ancestry), Latin America, Middle East, Africa, East Asia, South Asia, and South-East Asia (because the sex ratio in South-East Asian countries differs significantly from those in East Asia and South Asia). We also compared all those studies in which esotropia exceeds exotropia (Western countries, Middle Easterners, and North Indians) with ethnicities in which exotropia exceeds esotropia (mostly East Asians, South Indians, West Africans, and Persians). To examine correlations between societal attitudes towards gender and gender differences in strabismus clinics, we used the sex ratio as a proxy for societal attitude and calculated the *R^2^* in a regression model to see how much of the gender differences can be explained by societal or cultural attitudes. Similarly, we used a simple linear regression model to test the effects of socioeconomic factors (gross domestic product (GDP) per capita or income level, World Bank, 2016-2020) on gender differences in the clinics.

## RESULTS

**Figure 2:**
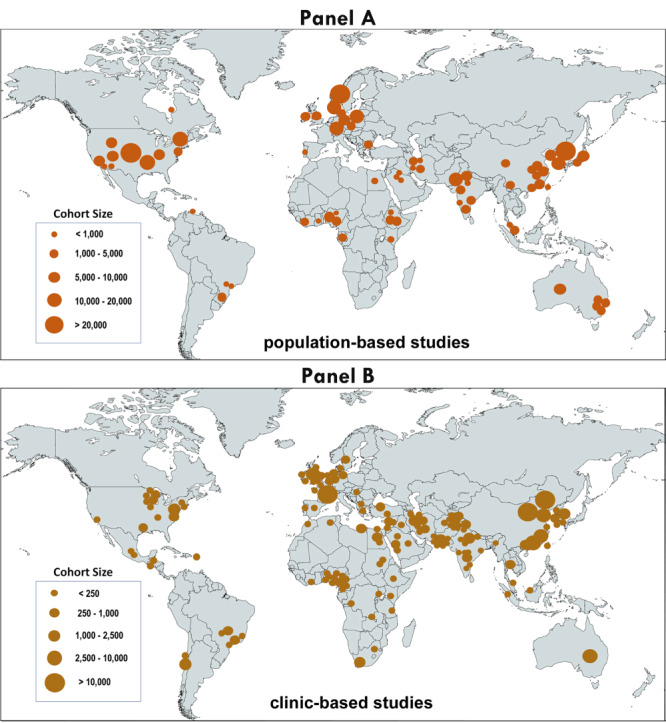
World maps showing the distribution and sizes of cohorts for studies on horizontal strabismus with gender information. **Panel A**. For 73 population-based studies. **Panel B**. For 141 clinic-based studies. The circles representing each cohort were placed over the study site. When the cohorts were drawn from multiple locations within the same country, the circle was placed in the centre of the various locations, e.g. Western and Central territories for indigenous Australians (panel A), metropolitan areas of Australia (panel B).

We found 739 records with quantitative information on horizontal strabismus, 427 were population-based, and 312 were clinic-based (**Figure 1**). Among the 427 population-based studies that reported on the prevalence of horizontal strabismus, 74 (17.33%) disclosed the distribution by gender in cohorts and in cases [10,14-16,22,25,32-98]. One of these studies was removed because it exclusively examined people above the age of 50 years [63]. The remaining 73 population-based studies provided information on a total of 384 658 subjects (194 944 males and 189 664 females; strabismus cases: 4980 males and 4890 females). Partially overlapping with these reports, 42 studies provided gender information about esotropia (cases: 1979 males and 1911 females), and 43 studies provided gender information on exotropia (cases: 1477 males and 1447 females) [10,14-16,22,32-35,37,41,43,44,46-54,57,61,62,64,65,67,69-71,74,77-79,82,84,85,90-93]. The global distribution of the 73 population-based studies reporting on horizontal strabismus is shown, along with their cohort sizes, in **Figure 2**, panel A. One study only reported males and was included in the summary statistics but not in the meta-analysis of gender proportions [54].

Among the 312 clinic-based studies that reported on clinic-visits for people with horizontal strabismus, 149 (47.76%) disclosed the gender distribution [1-9,11-13,18-21,99-230]. Seven studies [127,137,140,168,204-206] reported exclusively about 50+ year-old adults. Since we are interested in the age group that is comparable to that in the population-based studies, these seven studies [127,137,140,168,204-206] were removed. One study [195] was exclusively based on patients with insurance; it was also removed from the meta-analysis because none of the other studies disclosed insurance status. A total of 141 clinic-based studies were included in the meta-analyses (**Table 1**). The global distribution of the 141 studies and their cohort sizes is shown in **Figure 2**, panel B. After adjustment of the female numbers to take into account the unequal sex ratio in the general population, there were 78 442 males and 85 732 females [1-9,11-13,18-21,99-126,128-136,138,139,141-203,207-230]. Among the clinic-based studies, 40 studies detailed gender information for esotropia (4983 males and 5526 females, adjusted for the sex ratio) [4,12,13,20,99,101,104-107,111,115,119,122,132,133,136,139,143,144,146,150,154,160,163,164,167,174,176,180,187,198,200,209,211,213,219-221,229], and 51 studies detailed gender information for exotropia (4056 males and 5122 females, adjusted for the sex ratio) [1,4,6,8,9,12,13,19-21,99,104-107,111,112,115,117,119,122,128,132,133,136,139,143,144,150,154,160,162-164,174,176,180,184,194,198,200,201,209,211,213,217-221,229].

**Table 1:** Gender bias in clinic-based studies for horizontal strabismus based on a compilation of raw data on studies, cohorts, and summary statistics

Ethnicity/geography (all horizontal strabismus)	All horizontal strabismus: female bias	Esotropia	Female bias	Exotropia	Female bias
South Asia – 25 studies	1.55%	8 studies†	-7.90%	10 studies†	1.43%
*Male 3411*		*Male 433*		*Male 491*	
Female 3464*		Female 399*		Female 498*	
East Asia – 14 studies	8.18%	1 study	-6.10%	3 studies	12.67%
*Male 33 350*		*Male 29*		*Male 1 247*	
Female 36 079*		Female 27*		Female 1405*	
Western countries – 23 studies	9.09%	10 studies	14.85%	14 studies	55.80%
*Male 34 219*		*Male 1879*		*Male 1052*	
Female 37 329*		Female 2158*		Female 1639*	
Middle East – 17 studies	13.95%	7 studies	4.31%	7 studies	17.15%
*Male 2516*		*Male 649*		*Male 274*	
Female 2867*		Female 677*		*Female 321*	
South-East Asia – 3 studies	15.88%	0 studies		1 study	333.33%
*Male 233*		*n.d.*		*Male 3*	
Female 270*		*n.d.*		Female 10*	
Africa – 27 studies	17.84%	11 studies	16.22%	13 studies	6.96%
*Male 3083*		*Male 1338*		*Male 776*	
Female 2370*		Female 1555*		Female 830*	
Latin America – 12 studies	26.94%	3 studies	8.76%	3 studies	96.71%
*Male 1867*		*Male 628*		*Male 213*	
Female 2370*		Female 683*		Female 419*	
Global – 121 studies	9.29%	40 studies†	12.38%	51 studies†	26.28%
*Male 78 442*		*Male 4983*		*Male 4056*	
Female 85 732*		Female 5526*		Female 5122*	

n.d. – no data

*Numbers of females are adjusted for sex ratio, 0-14 years age range, according to the United Nations [28].

†There is partial overlap between studies reporting “horizontal strabismus” and studies that distinguish esotropia and exotropia. Therefore, percentages and numbers in the columns “esotropia” and “exotropia” do not add to the total in the column “All horizontal strabismus”.

### The prevalence of horizontal strabismus

**Figure 3:**
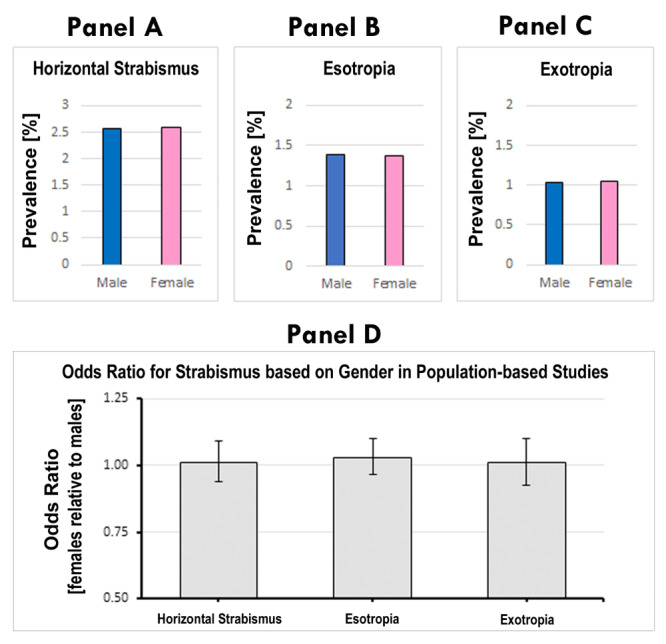
Lack of gender difference in horizontal strabismus, according to population-based studies. **Panel A**. Based on summary statistics of 73 studies with gender information, the prevalence of horizontal strabismus was 2.56% for males and 2.58% for females. **Panel B**. Based on summary statistics of 42 population-based studies, the prevalence for esotropia was 1.39% for males, and 1.38% for females. **Panel C**. According to 43 studies, the prevalence of exotropia was 1.03% for males, and 1.04% for females. **Panel D**. Meta-analysis results show that the odds ratios for gender in horizontal strabismus, esotropia, and exotropia range between OR = 1.01 and 1.03, with 95% CI = 0.94-1.09, 0.97-1.10, and 0.92-1.10, respectively; none of them reveal a statistically significant difference. The error bar indicates the 95% confidence interval.

We found that for overall horizontal strabismus, males had a prevalence of 2.558%, while females had a prevalence of 2.582%, based on 194 080 males and 188 922 females in these cohorts (**Figure 3**, panel A). According to our meta-analysis, this gender difference was statistically insignificant, with an OR = 1.01; 95% CI = 0.94-1.09, *P* = 0.824 (Figure S1 in the **Online Supplementary Document)**. A funnel plot did not show evidence of asymmetry (*P* = 0.162), indicating a lack of publication bias (Figure S3, panel A in the **Online Supplementary Document**).

According to the studies that detailed gender information specifically for esotropia, the prevalence of esotropia was 1.386% for males (n = 1979) and 1.377% for females (n = 1911), a difference between genders that was statistically insignificant (OR = 1.03; 95% CI = 0.97-1.10, *P* = 0.338). Based on studies that provided information specifically on exotropia, the prevalence of exotropia was 1.035% for males (n = 1477) and 1.043% for females (n = 1447); again, this gender difference was statistically insignificant (OR = 1.01; 95% CI = 0.92-1.10, *P* = 0.838). We conclude that there are no significant male-female differences in the prevalence of horizontal strabismus, neither for esotropia nor for exotropia (**Figure 3**, panels B, C and D).

The number of subjects for “overall” horizontal strabismus and the sum for esotropia and exotropia are not identical because many studies reported only aggregates and did not distinguish between esotropia and exotropia among horizontal strabismus cases. It should be noted that the prevalence we report (for horizontal strabismus, esotropia, and exotropia) are based on the studies that reported gender information; these studies are not necessarily representative of the entire global population and the population sizes of different ethnicities. Our data set appears to be Eurocentric, with fewer studies from Asian populations, many of which have a larger prevalence of exotropia than esotropia – which is not reflected by our study that focuses on gender distribution.

We examined whether the gender distribution differed between populations where exotropia was more frequent than esotropia (n = 33) compared with studies where esotropia was more frequent (or the same) as exotropia (n = 40). The prevalence was 2.42% for males in the former studies and 2.41% for females in the latter set of studies, which is no significant difference. Neither was there a gender difference in the studies where esotropia exceeded exotropia (or was identical): 2.67% for males and 2.71% for females; again, there was no statistically significant difference between genders.

### Females are more frequent than males in clinic-based studies of strabismus

Is the frequency with which patients seek medical attention for horizontal strabismus significantly different between males and females? And are there any differences in this respect between esotropia and exotropia? Clinic-based studies cannot report strabismus prevalence – only the frequency by which subjects seek medical attention for horizontal strabismus [231,232]. When gender information is provided, such studies indicate whether males and females visit clinics with the same frequency.

Six clinic-based studies exclusively examined subjects 50+ years of age [137,140,168,204-206]. We excluded these studies on older age so the remaining studies could be directly compared with the population-based studies as explained in the Methods.

**Figure 4:**
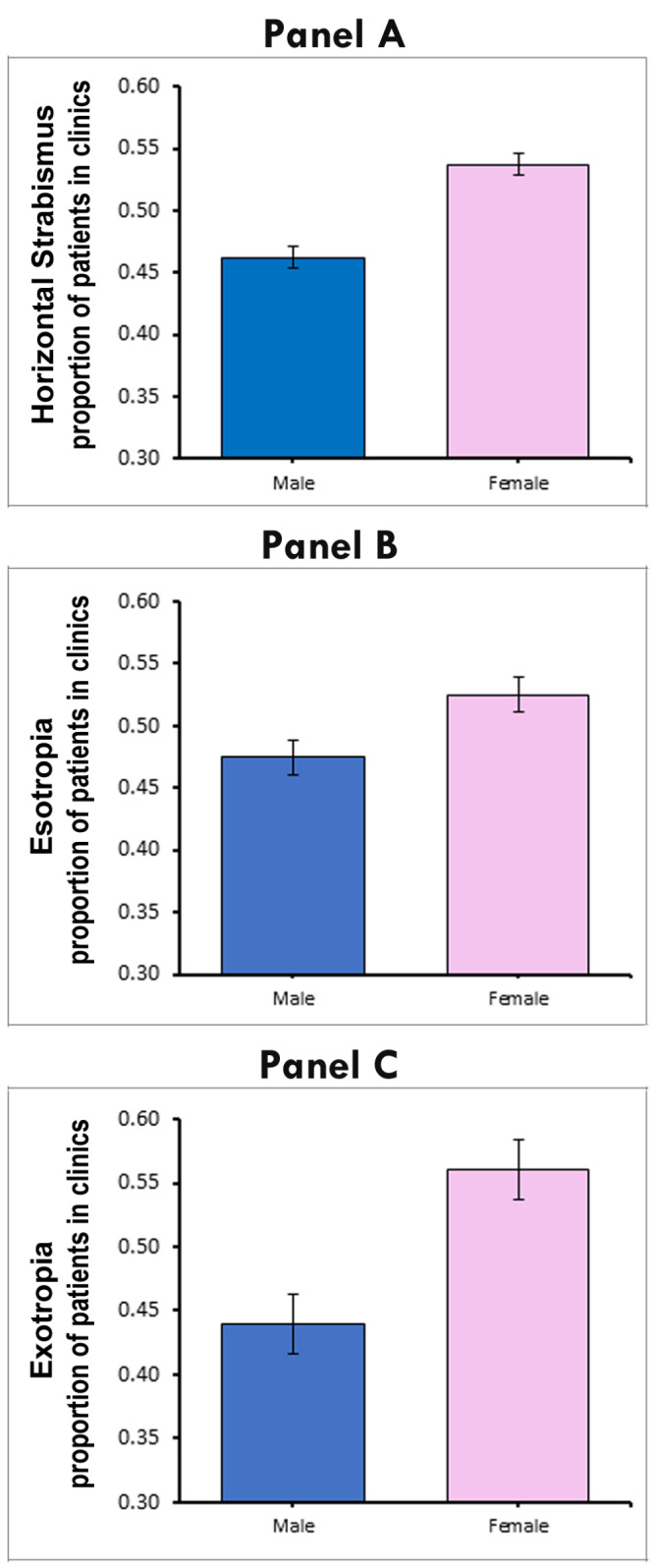
**Panel A.** Significantly more females than males visit clinics for horizontal strabismus. Based on 141 clinic-based studies, the proportion of females is 0.5375 vs. 0.4625 for males, a statistically significant difference of 7.50%. **Panel B**. Based on 40 studies for esotropia, 5.00% more females than males visit strabismus clinics. **Panel C**. Based on 51 studies for exotropia, 12.20% more females than males visit strabismus clinics. The error bars indicate the 95% confidence intervals.

For horizontal strabismus globally, females were seen more frequently in the clinic than males (**Table 1**, **Figure 4**, panel A). When adjusted for the sex ratio in the general population, the random effects model gives a female proportion of 0.54 (95% CI = 0.53-0.55, *P* < 0.001), and a male proportion of 0.46 (95% CI = 0.45-0.47, *P* < 0.001). The metaprop analysis thus reveals a 7.50% total difference between male and female clinic visits for horizontal strabismus, which is similar to the summary statistics (raw data) of a 9.29% gender difference (**Table 1**). This means that females seek medical attention for this condition significantly more often than males. The funnel plot (Figure S3, panel B in the **Online Supplementary Document**) did not show evidence of asymmetry (*P* = 0.786), indicating a lack of publication bias.

To determine whether both esotropia and exotropia contribute to the gender difference in the clinic-based studies, we analysed the studies that disclosed gender information specifically for these types of horizontal strabismus. There were 40 studies with 4983 males and 5526 females (adjusted for the sex ratio) for esotropia, and 51 studies with 4056 males and 5122 females (adjusted for the sex ratio) for exotropia. For the esotropia studies, we found that 5.00% more females than males were present in the clinic, a difference that was not statistically significant (*P* = 0.166). For the exotropia studies, we found that 12.20% more females than males were present in the clinic, which was a statistically significant difference (*P* < 0.001). Accordingly, the gender imbalance is more pronounced for exotropia than for esotropia (**Figure 4**, panels B and C). The raw data (summary statistics) shown in **Table 1** confirm that the gender difference for exotropia is approximately 2-fold higher than for esotropia. These percentages differ from the percentages of overall horizontal strabismus because there is only a partial overlap between the studies reporting on overall strabismus and the smaller number of studies reporting on esotropia and exotropia separately. Since the prevalence of horizontal strabismus is the same between males and females, the gender difference seen in clinic-based studies cannot result from gender differences in the prevalence. The larger gender bias in exotropia than in esotropia is seen in nearly all ethnicities (**Table 1**).

### Subgroup analyses: Differences between ethnicities or geographic regions

Is there a significant difference, or are there trends, in the above comparisons between major ethnicities or geographic regions with different societal attitudes towards genders? The female gender bias is evident in both types of populations: those that have a higher prevalence of esotropia than exotropia but also in populations that have a higher prevalence of exotropia than esotropia. This indicates that the type of horizontal strabismus that is predominant in the population is not what is driving the gender bias. Female bias was seen when all studies where esotropia exceeded exotropia were compared to those studies where exotropia exceeded esotropia (10.37% vs. 8.26%, not a significant difference, two-proportion test, *P* = 0.054).

**Figure 5:**
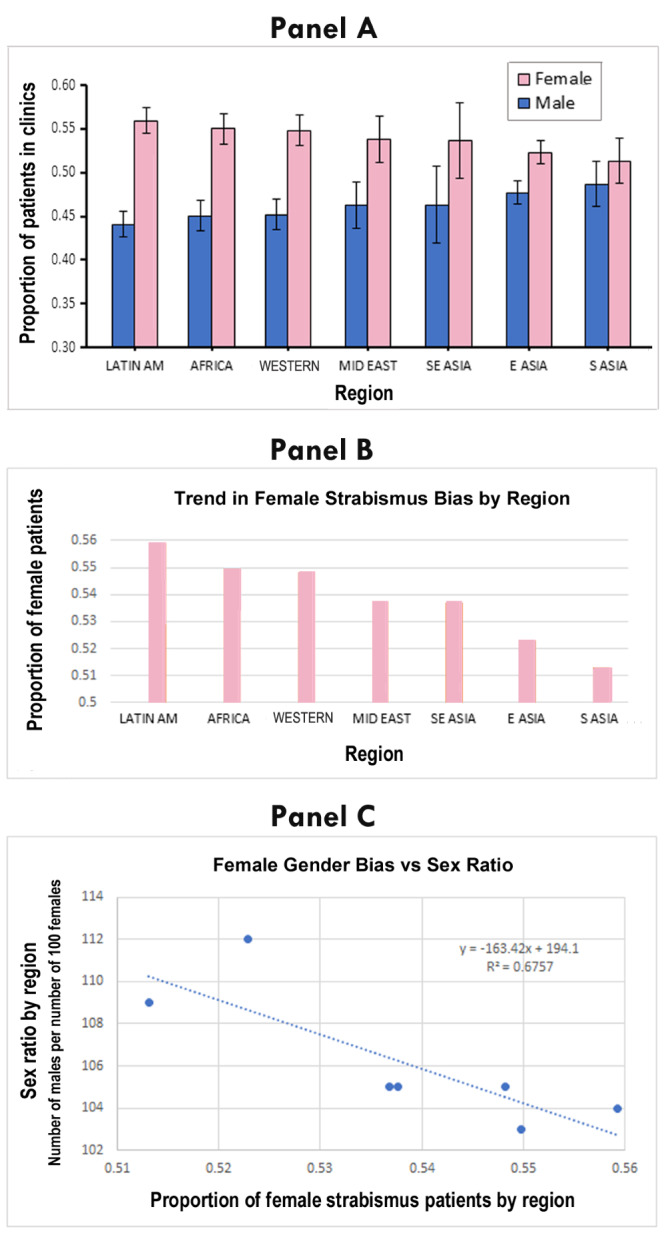
Female bias in health care utilisation for horizontal strabismus shows a trend between regions/societies that strongly associates with the extent of the sex ratio in those regions. **Panel A**. The difference in the proportion of males vs. females is most pronounced in Latin America (Latin Am), and is least in South Asia (S Asia). Bars represent the 95% confidence intervals. **Panel B**. The female bias is highest in Latin America, Africa, and Western countries and the least in Asian countries. **Panel C**. This trend is strongly and negatively associated with the sex ratio in these regions, expressed by the number of males per number of 100 females in the age range zero to 14 years [28]. The association has an *R^2^* value of 0.68, meaning that the model explains more than two thirds of the variation between regions.

The female gender bias is consistent across different ethnicities and regions. However, the female bias shows a gradient, with most Asian regions having a much smaller female bias than the other regions (**Table 1**, **Figure 5**, panels A and B). The extent of the gender difference for all horizontal strabismus between regions is mirrored by the sex ratio. When the extent of female gender bias in strabismus clinics is plotted against the sex ratio in the general population, there is a relatively strong negative correlation (**Figure 5**, panel C). The low female gender bias in South Asia and East Asia correlates negatively with the exceptionally high male-dominated sex ratio in these regions (*R^2^* = 0.676, *P* < 0.001). This indicates that societal gender biases play a role in the observed gender differences. Our linear regression model showed no correlation of gender bias with socioeconomic parameters such as GDP per capita (*R^2^* = 0.104, *P* = 0.260) or low-income vs. high-income status (*R^2^* = 0.078, *P* = 0.332).

### Subgroup analysis for generations

Are there differences or trends in the gender bias between generations? We examined this for Western countries only, because records are lacking or are too sparse for previous generations in other ethnicities. For populations of mostly European ancestry, the first three generations (1900-1970) showed a pooled female gender bias of 7.0%, while the last two generations (1971-2022) showed a pooled gender bias of 9.2%. In the generations that we were able to reliably analyse (a larger number of studies and cohorts), the difference between gender biases was statistically not significant (*P* = 0.190). We conclude that there is no evidence for clearly discernible trends over the last five generations.

## DISCUSSION

Our systematic review and meta-analysis examines gender differences in horizontal strabismus and also compares such gender differences between population-based and clinic-based studies. We resolve the controversy of whether or not there is a higher prevalence of exotropia in females than males: we found that there is no difference in the prevalence of horizontal strabismus between genders, neither for esotropia nor for exotropia. The erroneous notion of such a gender difference was based almost exclusively on clinic-based studies – yet results from clinic-based studies cannot be extrapolated to a population-based design, and prevalence cannot be inferred because of selection bias (bias towards people who are more concerned or distressed by their condition) in clinic-based studies [231,232]. Clinic-based studies do not report the prevalence, only the relative frequency at which patients seek medical attention. Another reason for the erroneous notion of a gender difference may have been the reliance on studies with small cohorts. Our systematic review was designed to minimise publication bias (by considering reports written in 22 different languages, including theses, technical reports, and conference proceedings; see Methods). Indeed, our funnel plots (Figure S3, panels A and B in the **Online Supplementary Document**) do not indicate publication bias. Consistent with the expectation of larger variation with smaller cohorts, the population-based studies with the larger cohorts [14-16] generally show the smallest gender bias or no gender difference at all.

Our most surprising finding is that there is a large and consistent gender difference in the frequency with which medical attention is sought for horizontal strabismus. Females are diagnosed and/or treated significantly more often for horizontal strabismus than males, even though the prevalence is the same for males and females. Within the field of vision and ophthalmology, this phenomenon appears to be relatively specific for strabismus since there is no such female bias for patients seeking medical attention for other vision conditions [233-235]. In fact, there is a large and globally consistent gender bias towards males for the treatment of eye disorders such as cataract (reviewed in [236]), similar to many other medical conditions [237]. Why is there such a large difference between males and females seeking medical attention for horizontal strabismus? Several potential reasons need to be considered.

Are females more attentive to horizontal deviations of the visual axis? Apparently, females do not detect strabismus more easily than males [238], so this does not explain the large gender difference, although females have greater expectations for corrective surgery than males [239].

Do females have a greater deviation of the visual axis than males so that female strabismus is easier to notice than male strabismus? While deviations of more than 40 degrees generate more concern among parents than deviations of less than 40 degrees [240], some studies have reported that males had greater deviation angles than females [240]. The interpupillary distance is generally shorter in females, and that should make it more difficult to detect strabismus in females than in males [238].

Are the consequences of strabismus more severe for females? There is a gender difference in horizontal strabismus in finding employment [239,241]. Likewise, a gender difference has been reported for finding a partner [242,243]. Whether esotropia or exotropia is “worse” in this respect is somewhat controversial. Exotropia was considered more detrimental for dating and employment than esotropia [239]. But for males, one study reported that esotropia was worse than exotropia [244].

Are parents more worried about strabismus in their daughters than in their sons? Indeed, several studies indicate that parents are more concerned about the looks of their daughters than their sons [11,23,25]. Since strabismus is mostly diagnosed and treated in childhood, this points to the parents as the decision makers and to the contribution of the parents as a source of the gender bias. Parents seem to intuitively “know” that strabismic appearance is more detrimental in females (girls are supposed to be beautiful) than in males (where earning potential is more important), so strabismic females are more disadvantaged for finding a partner and employment, as discussed above. Overall, this could explain why parents are more worried about the consequences of strabismus in daughters than in sons. If true, this shows the consequences of the notion among laypeople that strabismus is merely a cosmetic problem and obviously calls for enhanced educational efforts [245].

The gender bias does not seem to be specific for certain generations, at least not in Western populations. Apparently, this is a profound, ubiquitous and enduring phenomenon. The magnitude of the female bias in horizontal strabismus in the clinic appears to be affected by societal parameters because the gender bias shows significant association with other region-specific parameters of gender biases and mindsets. The effect is notably lower in Asian populations – likely due to societal attitudes, values and traditions in East and South Asia [27,246]. By contrast, the female gender bias is particularly strong in Latin America – possibly reflecting this region’s notable valuation of (female) beauty, as demonstrated by a large body of literature, studies, and statistics [247-249], including the fact that Latin American countries rank at the top of the global statistics for per capita cosmetic procedures [247].

Why is the gender bias so much larger for exotropia (12.20%) than for esotropia (5.02%)? Exotropia is more easily detectable and noticeable than esotropia. An outwards deviation can be detected when the misalignment is as little as six to nine prism dioptres (D), while detection of an inwards deviation requires nine to 12 D [239,250-252]. This could explain why exotropia is more often a concern than esotropia (**Table 1**, **Figure 4**) – it is more noticeable to parents. This again points to the parents as a major reason for the gender bias that is more pronounced with exotropia than with esotropia. Furthermore, recent studies showed that there are differences in the detection of strabismus between ethnicities [239,252]. However, the relatively small female gender bias in Asia (as compared with all other regions) is most likely explained by cultural differences in the valuation of genders [27,237,246].

Interestingly, the female bias seems to be reduced by about one-half when all the patients have health insurance (female bias is about 4%), compared to similar studies where the insurance status is unknown (female bias of about 9%) [195]. Lack of affordability is a known barrier to health care utilisation in paediatrics [253]. This indicates that economic factors, besides societal attitudes, play a role in the magnitude of female bias.

The strengths of our study are that it is based on a large number of studies and subjects. Limitations are that relatively few studies are available that distinguish gender data for esotropia and exotropia. Also, sparse data are available for some populations, e.g. native Americans and South-East Asians. The affordability of health care as a factor in gender bias requires more attention. We noticed that insurance status is rarely disclosed – the role of insurance for gender bias remains to be explored and verified. We applied sex ratio adjustments as reported for entire countries, but we did not attempt to adjust the ratio for regional differences within larger countries [27].

## CONCLUSIONS

We have discovered and documented an example of systemic discrimination of the male, rather than the female gender, apparently due to parent’s primary concerns about the cosmetic consequences of horizontal strabismus for female children, resulting in relative neglect of male children.

## Additional material


Online Supplementary Document
