# Supplementary material for: Gender differences in horizontal strabismus: Systematic review and meta-analysis shows no difference in prevalence, but gender bias towards females in the clinic
Source: J Glob Health. 2023 Sep 1;13:04085. doi: 10.7189/jogh.13.04085 (PMC10471156; doi:10.7189/jogh.13.04085)
Supplement: Online Supplementary Document [file jogh-13-04085-s001.pdf]

Online Supplementary Document – Laughton et al. Gender Differences

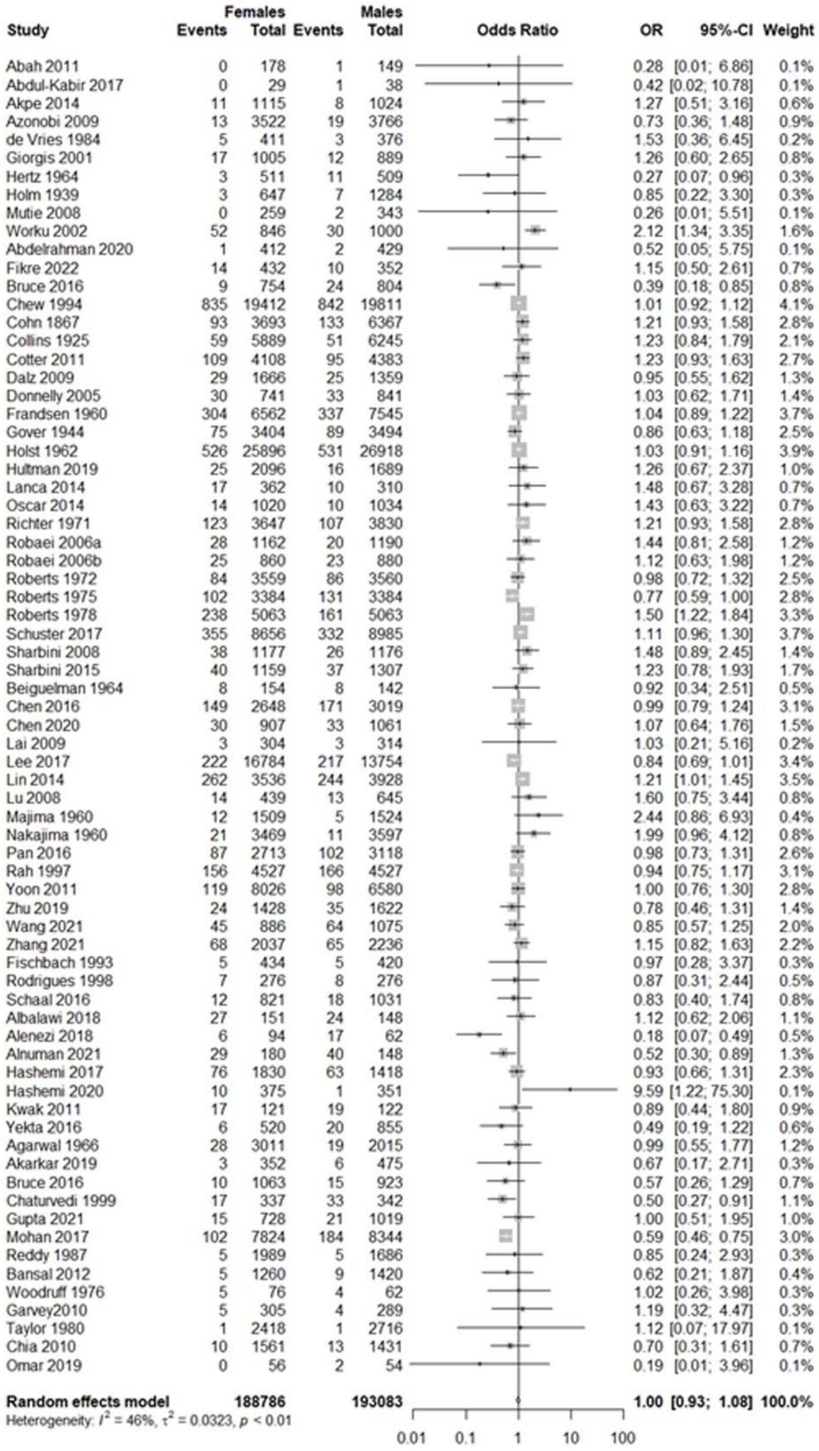

Figure S1. Forest plots of the odds ratio (OR) for males vs females in population-based studies of horizontal strabismus according to the meta-analysis. The OR is not significantly different between males and females (OR = 1.008, 95% confidence interval (CI) = 0.937-1.086; p=0.8244). The study weights are obtained based on the DerSimonian-Laird method. CI, confidence interval; DL, DerSimonian-Laird method; I^2, I-squared index.

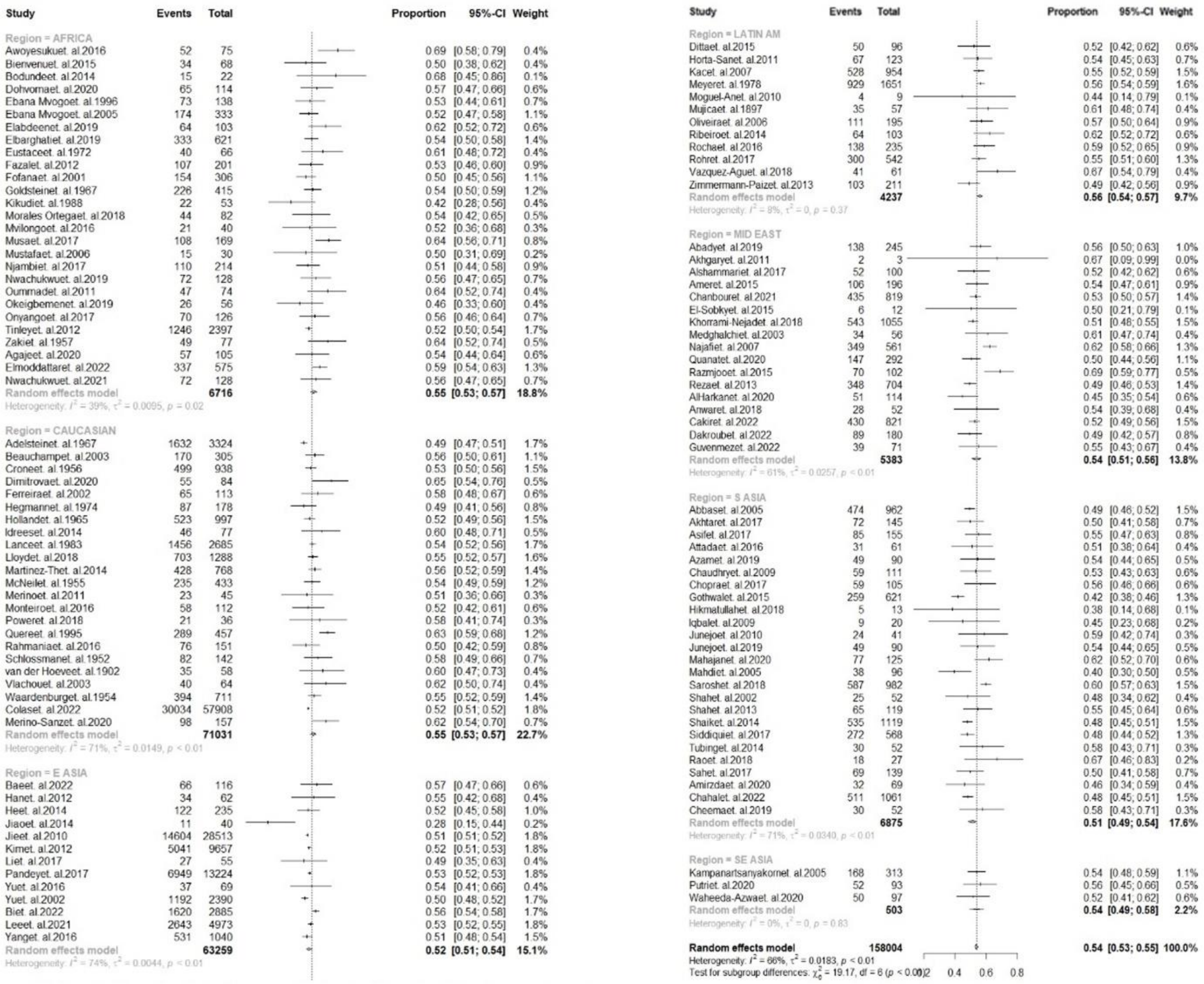

Figure S2. Forest plot for overall horizontal strabismus: clinic-based studies (n=121). Grouped by region/ethnicity (Africa, Caucasian, East Asia, Latin America, Middle East, South Asia, South-East Asia). Note the female bias, less pronounced in Asian countries. Globally, the random effects model gives a female proportion of 0.5375 (CI=0.5287; 0.5463, p<0.0001), and a male proportion of 0.4625 (95% CI= 0.4537; 0.4713, p<0.0001).

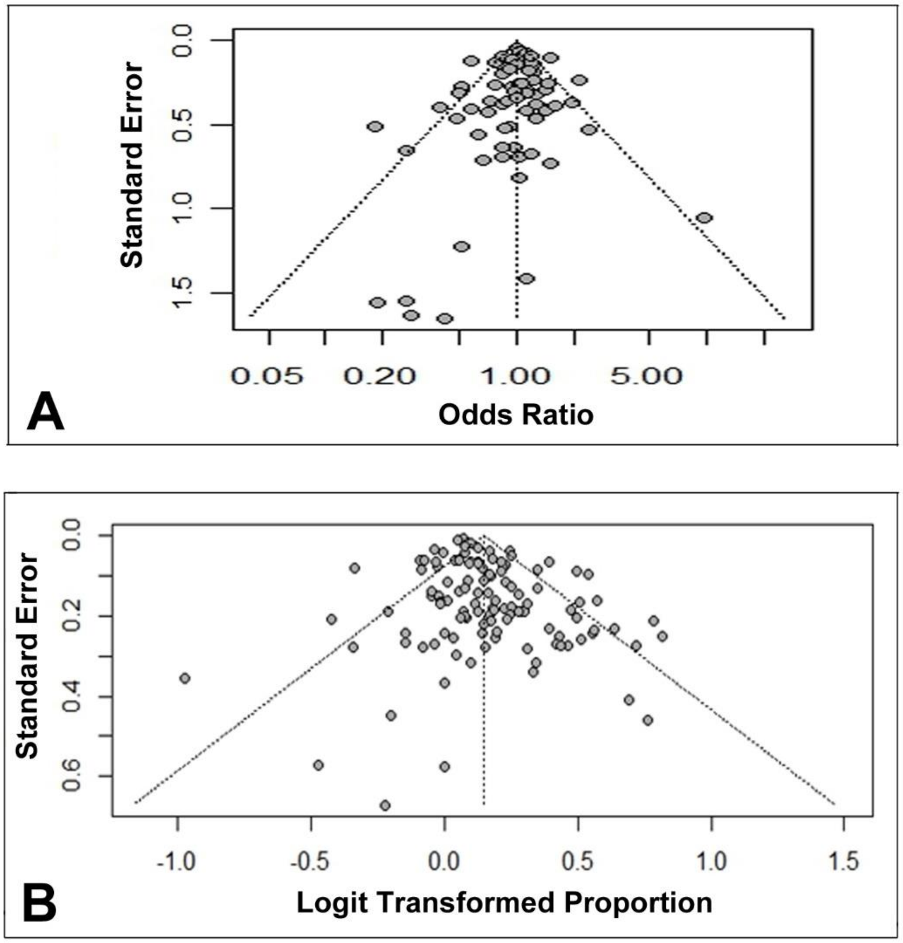

Figure S3A-B. Funnel plots. **A.** The funnel plot of 72 population-based studies on horizontal strabismus shows no evidence for asymmetry (rank correlation test, z = -1.40, p=0.1615), which means that publication bias is unlikely. **B.** The funnel plot of 121 clinic-based studies on horizontal strabismus shows no evidence of asymmetry (rank correlation test, z = 0.27, p=0.7863; p=0.7837 with one outlier removed), meaning that publication bias is unlikely. Rank correlation test was performed according to Begg and Mazumdar, 1994, Biometrics 50, 1088-1101.

SUPPLEMENTAL TABLES

Supplemental Table S1. All references disclosing gender (in cohorts as well as the cases) for population-based studies on horizontal strabismus (n=73) are listed alphabetically by author - excluding studies focusing on ages 50+ years.

---

1. Abah ER, Oladigbolu KK, Samaila E, Gani-Ikilama A. Ocular disorders in children in Zaria children's school. Niger J Clin Pract. 2011; 14(4):473-476.
2. Abdelrahman A, Abdellah M, Alsamman A, Radwan G. The prevalence of strabismus in children at school age in Sohaf City. 2020; Egypt J Clin Ophthalmol 3(1):11-17.
3. Abdul-Kabir M, Abdul-Sadik A, Ansah DO and Ofosu-Koranteng L. Prevalence of anisometropia, strabismus and amblyopia among first year optometry students in Kwame Nkrumah University of Science and Technology, Ghana. Mathews J Ophtalmol. 2017; 2(2):018.
4. Agarwal LP, Prakash P, Mahur A, Pathak MM. Visual defects in school children. Orient Arch Ophthal. 1966; 4:1-8.
5. Akarkar SO, Naik PG, Cacodcar JA. Prevalence and distribution of ocular morbidities among primary school children in Goa. J Clin Ophthalmol Res 2019; 7:61-64.
6. Akpe BA, Dawodu OA, Abadom EG. Prevalence and pattern of strabismus in primary school pupils in Benin City, Nigeria. Nigerian J Ophthalmol. 2014; 22:38-43.
7. Albalawi AMA, Alharbi AAH, Alanazi FHA, Alahmari HSH, Alharthi MFM, AlNasser HHT, Alrajoudi RAI, Lami AIM, Almutairi JAH, Alshammari ASS, Alnosair AO. Pattern of strabismus in children and adolescents in Hail, KSA. J Health Med Nursing. 2018; 54:28-33.
8. Alenezi HM, El-Fetoh NMA, Alruwaili AS, Alanazi WL, Alanazi NSF, Alanazi MSF, Alanazi SL, Alanazi TS, Alanazi BAS, Alanazi OF, Alrwaili AA. Squint in children and adolescents, Arar, Northern Saudi Arabia. Egypt J Hops Med. 2018; 70(2):298-302.
9. Alnuman RWS, Alhablani FSN, Alruwaili EMA, Alruwaili GAA, Alruwaili RR, Alruwaili RMJ, Zaky KAE. Prevalence of squint in primary school children and its associated sociodemographic factors in Sakaka City, Aljouf Region, Saudi Arabia. Int J Med Devel Countr 2021; 5(4): 1085-1091.
10. Azonobi IR, Olatunji FO, Addo J. Prevalence and pattern of strabismus in Ilorin. West Afr J Med. 2009a; 28(4):253-256.
11. Bansal A, Krishnappa K, Datti NP, Guruprasad BS, Guha J. Ocular morbidity in school going children of Kolar district, South India. J Clin Biomed Sci 2012; 2(4): 175-184.
12. Beiguelman B. A survey on genetical and anthropological traits among Japanese immigrants in Brazil. Z Morphol Anthropol. 1964; 55(1):46-59.
13. Bruce A, Santorelli G. Prevalence and risk factors of strabismus in a UK multi-ethnic birth cohort. Strabismus. 2016; 24(4):153-160.
14. Chaturvedi S, Aggarwal OP. Pattern and distribution of ocular morbidity in primary school children of rural Delhi. Asia-Pacific J Public Health 1999; 11(1), 30-33.
15. Chen X, Fu Z, Yu J, Ding H, Bai J, Chen J, Gong Y, Zhu H, Yu R, Liu H. Prevalence of amblyopia and strabismus in Eastern China: results from screening of preschool children aged 36-72 months. Brit J Ophthalmol. 2016; 100(4):515-519.
16. Chen D, Li R, Li X, Huang D, Wang Y, Zhao X, Zhang X, Sun Q, Hao Q, Tong H, Yao X, Fan W, Lu W, Dang J, Zhu H, Liu H. Prevalence, incidence and risk factors of strabismus in a Chinese population-based cohort of preschool children: the Nanjing Eye Study. Br J Ophthalmol. 2021; 105(9):1203-1210.

17. Chew E, Remaley NA, Tamboli A, Zhao J, Podgor MJ, Klebanoff M. Risk factors for esotropia and exotropia. Arch Ophthalmol. 1994; 112(10):1349-1355.
18. Chia A, Dirani M, Chan YH, Gazzard G, Au Eong KG, Selvaraj P, Ling Y, Quah BL, Young TL, Mitchell P, Varma R, Wong TY, Saw SM. Prevalence of amblyopia and strabismus in young Singaporean Chinese children. Invest Ophthalmol Vis Sci. 2010; 51:3411-3417.
19. Cohn H. [Untersuchungen der Augen von 10060 Schulkindern nebst Vorschlaegen zur Verbesserung der den Augen nachtheiligen Schuleinrichtungen.] Leipzig, Verlag von Friederich Fleischer, 1867. https://archive.org/details/b2163693x
20. Collins SD. Strabismus and defective color sense among school children. Pub Health Rep. (Washington) 1925; 40:1515-1523.
21. Cotter SA, Varma R, Tarczy-Hornoch K, McKean-Cowdin R, Lin J, Wen G, Wei J, Borchert M, Azen SP, Torres M, Tielsch JM, Friedman DS, Repka MX, Katz J, Ibironke J, Giordano L; Joint Writing Committee for the Multi-Ethnic Pediatric Eye Disease Study and the Baltimore Pediatric Eye Disease Study Groups. Risk factors associated with childhood strabismus: the multi-ethnic pediatric eye disease and Baltimore pediatric eye disease studies. Ophthalmology. 2011; 118(11):2251-2261.
22. Dalz M, Gotz-Wieckowska A, Dalz M. [Schielenkrankheit bei den Kindern im Schulalter.] 107th Kongress, Deutsche Ophthalmologische Gesellschaft (DOG), Leipzig, Germany, Abstract P 094, 2009 https://2009.archiv.dog.org/abstracts/P094.html
23. De Vries B, Houtman WA, Van Bochove J. Prevalence of squint and amblyopia on the island of Curacao. Brit Orthopt J. 1984; 41:53-57.
24. Donnelly UM, Stewart NM, Hollinger M. Prevalence and outcomes of childhood visual disorders. Ophthalmic Epidemiol. 2005; 12(4):243-250.
25. Faghihi M, Ostadi moghaddam H, Fatemi A, Heravian Shandiz J, Yekta A. Strabismus and amblyopia in schoolboys of Varamin, Iran, in 2010. Iranian J Ophthalmol. 2012; 24:32-38.
26. Fikre B, Ferede AT, Tefera TK. Strabismus Prevalence and Associated Factors among Kindergarten School Children, Northwest Ethiopia. Int J Ophthalmol & Vis Sci. 2022; 7(2):45-50.
27. Fischbach LA, Lee DA, Englehardt RF, Wheeler N. The prevalence of ocular disorders among Hispanic and Caucasian children screened by the UCLA Mobile Eye Clinic. J Community Health. 1993; 18(4):201-211.
28. Frandsen AD. Occurrence of squint: a clinical-statistical study on the prevalence of squint and associated signs in different groups and ages of the Danish population. Acta Ophthalmol Suppl 1960; 62:9–157.
29. Garvey KA, Dobson V, Messer DH, Miller JM, Harvey EM. Prevalence of strabismus among preschool, kindergarten, and first-grade Tohono O'odham children. Optometry. 2010; 81(4):194-199.
30. Giorgis AT, Bejiga A. Prevalence of strabismus among pre-school children community in Butajira Town. Ethiop J Health Dev. 2001; 15(2):125-130.
31. Gover M, Yaukey JB. Physical impairments of members of low-income farm families; 11,490 persons in 2,477 Farm Security Administration borrower families, 1940; extent of immunization against smallpox, diptheria, and typhoid fever. Public Health Rep. 1946; 61:97-109.
32. Gupta PK, Caculo DU. The attitude towards strabismus and barriers for its treatment in parents from rural and urban areas. Indian J Clin Exp Ophthalmol 2021; 7(1):54-61.
33. Hashemi H, Nabovati P, Yekta A, Ostadimoghaddam H, Behnia B, Khabazkhoob M. The prevalence of strabismus, heterophorias, and their associated factors in underserved rural areas of Iran. Strabismus. 2017; 25(2):60-66.

34. Hashemi H, Pakzad R, Nabovati P, Azad Shahraki F, Ostadimoghaddam H, Aghamirsalim M, Pakbin M, Yekta A, Khoshhal F, Khabazkhoob M. The prevalence of tropia, phoria and their types in a student population in Iran. Strabismus. 2020; 28(1):35-41.
35. Hashemi H, Nabovati P, Yekta AA, Agha Mirsalim M, Rafati S, Ostadimoghaddam H, Khabazkhoob M. Binocular vision disorders in a geriatric population. Clin Exp Optom. 2022; 105(5):539-545.
36. Hertz J, Gombosh G, Avshalom A. Vision screening of students in Liberia. A preliminary report. J Pediatr Ophthalmol Strabismus 1964; 1(3):33-36.
37. Holm S. [Le strabisme concomitant chez les palénégrides au Gabon, Afrique Equatoriale Française. Contribution å la question de race et de strabisme.] Acta Ophthalmol. 1939; 17:367-387.
38. Holst JC, Tjaland J. [Some figures from the ophthalmological department of the schools in Oslo.] Tidsskr Nor Laegeforen. 1962; 82:1291-1293.
39. Hultman O, Beth Høeg T, Munch IC, Ellervik C, la Cour M, Andersson Grönlund M, Buch Hesgaard H. The Danish Rural Eye Study: prevalence of strabismus among 3785 Danish adults - a population-based cross-sectional study. Acta Ophthalmol. 2019; 97(8):784-792.
40. Kwak J, Movahedan J, Tadjvidi P, Aref P, Maumenee IH. Mendelian inheritance of strabismus in a rural community of Northern Iran. Invest Ophthalmol Vis Sci. 2011; 52(14):6359.
41. Lai YH, Hsu HT, Wang HZ, Chang SJ, Wu WC. The visual status of children ages 3 to 6 years in the vision screening program in Taiwan. J AAPOS. 2009; 13(1):58-62.
42. Lança C, Serra H, Prista J. Strabismus, visual acuity, and uncorrected refractive error in Portuguese children aged 6 to 11 years. Strabismus. 2014; 22(3):115-119.
43. Lee JF, Kim CZ, Nam KY, Lee SU, Lee SJ. [An epidemiological survey of strabismus and nystagmus in South Korea: KNHANES V.] J Korean Ophthalmol Soc. 2017; 58(11):1260-1268.
44. Lin S, Congdon N, Yam JC, Huang Y, Qiu K, Ma D, Chen B, Li L, Zhang M. Alcohol use and positive screening results for depression and anxiety are highly prevalent among Chinese children with strabismus. Am J Ophthalmol. 2014; 157(4):894-900.
45. Lu P, Chen X, Zhang W, Chen S, Shu L. Prevalence of ocular disease in Tibetan primary school children. Can J Ophthalmol. 2008; 43(1):95-99.
46. Majima A, Nakajima A, Ichikawa H, Watanabe M. Prevalence of ocular anomalies among school children. Am J Ophthalmol. 1960; 50:139-146.
47. Mohan A, Bisht A, Sharma VK, Jamil Z. Epidemiology of ocular morbidity among school-going children. All India Ophthalmol Soc Proc. 2017; FP1242.
48. Mutie DM. Ocular morbidity in nursery school children in Kilungu Division, Makueni District. Dissertation Master of Medicine, Ophthalmology, University of Nairobi, Kenya, 2008.
49. Nakajima A, Yoshimoto T, Ito N, Kimura T, Majima A, Awaya S. [Distribution of eye diseases among school children.] Rinsho Ganka (Jpn J Clin Ophthalmol). 1960; 14:1762–1769.
50. Omar R, Wan Abdul WMH, Knight VF. Status of visual impairment among indigenous (Orang Asli) school children in Malaysia. BMC Public Health. 2019; 19(Suppl 4):543.
51. Oscar A, Cherninkova S, Haykin V, Aroyo A, Levi A, Marinov N, Kostova S, Elenkov C, Veleva N, Chernodrinska V, Petkova I, Spitzer J. Amblyopia screening in Bulgaria. J Pediatr Ophthalmol Strabismus. 2014; 51(5):284-288.
52. Pan CW, Zhu H, Yu JJ, Ding H, Bai J, Chen J, Yu RB, Liu H. Epidemiology of intermittent exotropia in preschool children in China. Optom Vis Sci. 2016; 93(1):57-62.
53. Rah SH, Jun HS, Kim SH. [An epidemiological survey of strabismus among school-children in Korea.] J Korean Ophthalmol Soc. 1997; 38(12):2195-2199.

54. Reddy SC. Ocular morbidity and colour blindness among school children in Kakinada. The Antiseptic. 1987; 84(5): 611-616.
55. Richter J, Rosemann E, Rosemann U. [The morbidity of vision disorders in preschool children in Gorlitz]. Z Gesamte Hyg. 1971; 17(2):138-141.
56. Robaei D, Rose KA, Kifley A, Cosstick M, Ip JM, Mitchell P. Factors associated with childhood strabismus: findings from a population-based study. Ophthalmology. 2006a; 113(7):1146-1153.
57. Robaei D, Kifley A, Mitchell P. Factors associated with a previous diagnosis of strabismus in a population-based sample of 12-year-old Australian children. Am J Ophthalmol. 2006b; 142(6):1085-1088.
58. Roberts J. Eye examination findings among children, United States. Vital and health statistics: Series 11-No. 115, DHEW publication No. (HSM) 72-1057. Health Services and Mental Health Administration. Washington. U.S. Government Printing Office, June 1972.
59. Roberts J. Eye examination findings among youths aged 12-17 years, United States. Vital and health statistics: Series 11-No. 155, DHEW publication no. (HRA) 76-1637. Health Resources Administration. Washington. U.S. Government Printing Office, Nov. 1975.
60. Roberts J, Rowland M. Refractive status and motility defects of persons 4-74 years, United States 1971-1972. Vital and health statistics: series 11, DHEW publication no. (PHS) 78-1654. Hyattsville, MD: National Center for Health Statistics, 1978.
61. Rodrigues EB. [Estrabismo na criança - triagem em um ambulatorio de pediatria.] Dissertation, Universidade Federal de Santa Catarina, Florianopolis, Brazil, 1998.
62. Schaal LF, Schellini SA, Pesci LT, Galindo A, Padovani CR, Corrente JE. The prevalence of strabismus and associated risk factors in a Southeastern region of Brazil. Semin Ophthalmol. 2018; 33(3):357-360.
63. Schuster AK, Elflein HM, Pokora R, Urschitz MS. [Childhood strabismus in Germany: Prevalence and risk groups: results of the KiGGS survey]. Bundesgesundheitsblatt Gesundheitsforschung Gesundheitsschutz. 2017; 60:849e855.
64. Sharbini SH, Wang JJ, Burlutsky G, Rose KA, Mitchell P, Sydney Childhood Eye Study, Sydney Myopia Study. Parental factorts associated with strabismus in a population-based sample of 12-year old Australian children. Invest Ophthalmol Vis Sci. 2008; 49:1807 (abstr).
65. Sharbini SH. Prevalence of strabismus & associated risk factors: The Sydney Childhood Eye Studies. Dissertation, University of Sydney, Faculty of Health Sciences (Orthoptic), 2015, 290 pp.
66. Taylor HR. Prevalence and causes of blindness in Australian aborigines. Med J Aust. 1980; 1(2):71-76.
67. Wang Y, Zhao A, Zhang X, Huang D, Zhu H, Sun Q, Yu J, Chen J, Zhao X, Li R, Han S, Dong W, Ma F, Chen X, Liu H. Prevalence of strabismus among preschool children in eastern China and comparison at a 5-year interval: a population-based cross-sectional study. BMJ Open. 2021; 11(10):e055112.
68. Woodruff ME, Samek MJ. The refractive status of Belcher Island Eskimos. Can J Public Health. 1976; 67(4):314-320.
69. Worku Y, Bayu S. Screening for ocular abnormalities and subnormal vision in school children of Butajira Town, southern Ethiopia. Ethiop J Health Dev. 2002; 16(2):165-171.
70. Yekta A, Hashemi H, Ostadimoghaddam H, Haghighi B, Shafiee H, Mehravaran S, Nabovati P, Asharlous A, Khabazkhoob M. Strabismus and near point of convergence and amblyopia in 4-6 year-old children. Strabismus. 2016; 24(3):113-119.
71. Yoon KC, Mun GH, Kim SD, Kim SH, Kim CY, Park KH, Park YJ, Baek SH, Song SJ, Shin JP, Yang SW, Yu SY, Lee JS, Lim KH, Park HJ, Pyo EY, Yang JE, Kim YT, Oh KW, Kang SW. Prevalence of eye diseases in South Korea: data from the Korea National

Health and Nutrition Examination Survey 2008-2009. Korean J Ophthalmol. 2011; 25(6):421-433.
72. Zhang XJ, Lau YH, Wang YM, Kam KW, Ip P, Yip WW, Ko ST, Young AL, Tham CC, Pang CP, Chen LJ, Yam JC. Prevalence of strabismus and its risk factors among school aged children: The Hong Kong Children Eye Study. Sci Rep. 2021; 11(1):13820.
73. Zhu H, Pan C, Sun Q, Huang D, Fu Z, Wang J, Chen X, Wang Z, Liu H. Prevalence of amblyopia and strabismus in Hani school children in rural southwest China: a cross-sectional study. BMJ Open. 2019; 9(2):e025441.

Supplemental Table S2. All references disclosing gender for population-based studies on horizontal strabismus (n=141) are listed alphabetically by author, excluding studies focusing on ages 50+ years.

---

1. Abady NH, Al-jumaili AA, Fayyadh RA. Association between strabismus and refractive errors among preschool children in Fallujah, Iraq. Indian J Public Health Res Dev. 2019; 10(5):508-513.
2. Abbas S, Said A, Riad S. Incidence of the various types of squint: 1000 cases examined in the squint clinic Kasr El Aini Hospital. Bull Ophthalmol Soc Egypt. 1974; 67:311–314.
3. Adelstein AM, Scully J. Epidemiological aspects of squints. Br Med J. 1967; 3(5561):334-338.
4. Agaje BG, Delelegne D, Abera E, Desta K, Girum M, Mossie M, Eshetu D, Hirigo AT. Strabismus prevalence and associated factors among pediatric patients in southern Ethiopia: a cross-sectional study. J Int Med Res. 2020; 48(10):300060520964339.
5. Akhgary M, Ghassemi-Broumand M, Amiri MA, Seyed MT. Prevalence of strabismic binocular anomalies, amblyopia and anisometropia. Rehabilitation Faculty of Shahid Beheshti Medical University. J Optom. 2011; 4(3):110-114.
6. Akhtar N, Gupta S. Prevalence of types of strabismus in pediatric patients in a tertiary centre of North India. J Dent Med Sci. 2017; 16(6):61-64.
7. Alshammari M, Alhibshi N, Almusallam A, Badawood E, Abdulwassi H. Risk factors for developing different subtypes of strabismus in a Saudi population. Int J Med Health Res. 2017; 3(11):116-120.
8. Amer A. Relative Prevalence of Various Types of Strabismus in Patients Attending NGO's Medical Centers in Gaza Strip. Sci J Public Health. Special Issue: Health Behavior and Public Health. 2015; 3(1-1):1-5.
9. Amirzda SM. Prevalence of horizontal strabismus in pediatric patients at University Eye Hospital. J Opht Res Rev Rep 2020; 1(1):1-2.
10. Asif M, Habiba U, Khan MW, Kawish AB, Rehman A-u. Frequency of major types of manifest strabismus among patients of age group 1 to 25 years presented to Benazir Bhutto Hospital Rawalpindi. Pak J Med Biol Sci. 2017; 1:30-34.
11. Attada TR, Deepika M, Laxmi S. Strabismus in paediatric age (3-16 year): a clinical study. Int J Res Med Sci. 2016; 4(6):1903-1909.
12. Awoyesuku EA, Fiebai B, Onua AA. Pattern of strabismus in a tertiary hospital in Nigeria: a six-year review. Por Harcourt Med J. 2016; 10:14-17.
13. Azam S, Priyanka, Qasim M, Frequency of neurogenic strabismus in Al-Ibrahim eye hospital, Karachi. Pak. J. Ophthalmol. 2019; 35(2):122-126.

14. Beauchamp GR, Black BC, Coats DK, Enzenauer RW, Hutchinson AK, Saunders RA, Simon JW, Stager DR, Stager DR Jr, Wilson ME, Zobal-Ratner J, Felius J. The management of strabismus in adults--I. Clinical characteristics and treatment. J AAPOS. 2003; 7(4):233-240.
15. Bi Y, Yam JC, Lin S. A retrospective study of strabismus surgery in a tertiary eye hospital in the Chaoshan area in China from 2014 to 2020. BMC Ophthalmol. 2022; 22(1):246.
16. Bienvenu YA, Angel MN, Sebastien MM, Philippe CM, Léon KN, Eugene TK, Chami CL, Prosper KM, Claude SS, Gaby CB. [Study of strabismus in children 0-15 years followed in Lubumbashi, Democratic Republic of Congo: Analysis of epidemiological and clinical aspects]. Pan Afr Med J. 2015; 22:66.
17. Bodunde OT, Onabolu OO, Fakolujo VO. Pattern of squint presentations in children in a tertiary institution in Western Nigeria. J Dent Med Sci. 2014; 13(5):29-31.
18. Bristow JH, Douglas WHG, McCartney D, Swartz J, Armstrong EB, Schwyzer E. Divergent squint in Caucasoids. Cases seen in a paediatric hospital outpatients department. S Afr Med J. 1973; 47(40):1925–1926.
19. Çakır B, Aksoy NÖ, Bursalı Ö, Özmen S. Non-ocular risk factors in Turkish children with strabismus and amblyopia. Turk J Pediatr. 2022; 64(2):341-349.
20. Cass EE. Divergent Strabismus. Brit J Ophthalmol. 1937; 21(10):538-559.
21. Chahal V, Singh V, Singh J, Kaur G, Srivastava RM, Agrawal S. Profile of orthoptic clinic patients at a tertiary care Government Medical University in North India: A 6-year review. J Clin Ophthalmol Res. 2022; 10:27-32.
22. Chanbour H, Bsat A, Chanbour W, Cherfan C. Geographic Variation in Strabismus Pattern Among Pediatric Age Group in Lebanon: A Single-Centre Five-Year Observational Study. Cureus. 2021; 13(6):e15957.
23. Chang YS, Kim SY. [Clinical study of A-V pattern strabismus in Korea.] J Korean Ophthalmol Soc. 2008; 49(12):1974-1980.
24. Chaudhry TA, Khan A, Khan MB, Ahmad K. Gender differences and delay in presentation of childhood squint. J Pak Med Assoc. 2009; 59(4):229-231.
25. Cheema MN, Anwar S, Hussain A, Bangash MT, Afzal MFB, Shamas F. Frequency of Esotropia Among the Patients Presented with Ocular Misalignments Between the Age Group of 2-18 Years in Ophthalmology Outdoor Department at Islam Teaching Hospital. Pak J Med Health Sci 2019; 13(4):830-833.
26. Chen YW, Lin SA, Lin PW, Huang HM. The difference of surgical outcomes between manifest exotropia and esotropia. Int Ophthalmol. 2019; 39(7):1427-1436.
27. Chopra V, Balasubramanian P. Clinical study of concomitant squint. J Evid Based Med Healthc. 2017; 4(54):3294-3297.
28. Colas Q, Capsec J, Arsène S, Pisella PJ, Grammatico-Guillon L, Khanna RK. Strabismus outcomes after surgery: the nationwide SOS France study. Graefes Arch Clin Exp Ophthalmol. 2022; 260(6):2037-2043.
29. Crone RA, Velzeboer CM. Statistics on strabismus in the Amsterdam youth; researches into the origin of strabismus. AMA Arch Ophthalmol. 1956; 55(4):455-470.
30. Dakroub M, El Hadi D, El Moussawi Z, Ibrahim P, Al-Haddad C. Characteristics and long-term surgical outcomes of horizontal strabismus. Int Ophthalmol. 2022; 42(5):1639-1649.
31. Dawson E, Bentley C, Lee J. Squint surgery in the over sixties. Strabismus. 2001; 9(4):217-220.
32. Dharmaraju B, Vijayasree S, Mythili K. Study of aetiological factors contributing to paediatric strabismus. J Evid Based Med Healthc. 2016; 3(65):3520-3523.
33. Dimitrova-Radojichikj, Tasevska D. Causes of visual impairment: a retrospective study in Macedonian children. J Ophthalmol (Ukraine). 2020; 3(494):29-30.

34. Ditta LC, Pereiras LA, Graves ET, Devould C, Murchison E, Figueroa L, Kerr NC. Establishing a surgical outreach program in the developing world: pediatric strabismus surgery in Guatemala City, Guatemala. J AAPOS. 2015; 19(6):526-530.
35. Dohvoma VA, Ebana Mvogo SR, Mvilongo CT, Epee E, Ebana Mvogo C. Strabisme de l'enfance négligé: aspects épidémiologiques, cliniques et thérapeutiques [Neglected childhood strabismus: Epidemiological, clinical and therapeutic aspects]. J Fr Ophtalmol. 2020; 43(8):774-778.
36. Ebana Mvogo C, Bella-Hiag AL, Epesse M. [Strabismus in Cameroon]. J Fr Ophtalmol. 1996; 19(11):705-709.
37. Ebana Mvogo C, Ellong A, Owona D, Luma H, Bella LA. [Amblyopia and strabismus in our environment]. Bull Soc Belge Ophtalmol. 2005; 297:39-44.
38. Elabdeen RHZ, Ibrahim SM. Clinical study of vertical strabismus among patients attending squint clinic- Makkah Eye Hospital – Khartoum. Int Res Med Health Sci 2019; 2(5):1-19.
39. Elbarghathi AM, Elbarghathi MF, Abdullah RM. Epidemiology of Strabismus among Patients Visiting Squint Clinic in Benghazi. Appl Cell Biol. 2019; 7(1):6-11.
40. Elmoddather M. Prevalence of strabismus and its types in pediatric population and the outcomes of different treatment modalities: A 3-year prospective study in a referral eye center in Upper Egypt. Egypt J Hospital Med. 2022; 86:826-830.
41. El-Sahn MF, Granet DB, Marvasti A, Roa A, Kinori M. Strabismus in adults older than 60 years. J Pediatr Ophthalmol Strabismus. 2016; 53(6):365-368.
42. El-Sobky HM, Said Ahmed KE, El-Sawy MF, Sakr SM. Incidence of amblyopia in the Menoufia University outpatient clinic. Menoufia Med J. 2015; 28:207-213.
43. Eustace P. Myopia and divergent squint in West Indian children. Brit J Ophthalmol. 1972; 56(7):559-564.
44. Fang SY, Gandhi N, Satterfield D, O'Hara M. Strabismus surgery for Medicare-aged patients: more than a decade of insights. J AAPOS. 2018; 22(3):170-173.
45. Fazal AF. 2012. Review of outcome of horizontal childhood strabismus surgery at Kenyatta National Hospital and Kikuyu Eye Unit – a retrospective study. Dissertation, University of Nairobi, H58/78551.
46. Ferreira RC, Oelrich F, Bateman B. Genetic aspects of strabismus. Arc Bras Oftalmol 2002; 65:171-175.
47. Fofana Z. Approche medicale du strabisme au chu de Cocody. Thesis Doctorate en Meecine, Universite de Cocody, Abidjan, Cote d"Ivoire. 2001, 139 pp.
48. Goldstein H, Henderson M, Goldberg ID, Benitez E, Hawkins CM. Perinatal factors associated with strabismus in Negro children. Am J Public Health Nations Health. 1967; 57(2):217-228.
49. Gothwal VK, Bharani S, Kekunnaya R, Chhablani P, Sachdeva V, Pehere NK, Narasaiah A, Gunturu R. Measuring Health-Related Quality of Life in Strabismus: A Modification of the Adult Strabismus-20 (AS-20) Questionnaire Using Rasch Analysis. PLoS One. 2015; 10(5):e0127064.
50. Govindan M, Mohney BG, Diehl NN, Burke JP. Incidence and types of childhood exotropia: a population-based study. Ophthalmology 2005; 112:104-108.
51. Greenberg AE, Mohney BG, Diehl NN, Burke JP. Incidence and types of childhood esotropia: A population based study. Ophthalmology. 2007; 114(1):170-174.
52. Gregersen E. The polymorphous exo patient. Analysis of 231 successive cases. Acta Ophthalmol (Copenh). 1969; 47(3):579-590.
53. Guvenmez O, Kayiklik A. Strabismus in Pediatric Age: A Single Center Experience in Turkey. Ulutas Med J. 2019; 5(3):184-188. doi: 10.5455/umj.20191127100430
54. Han KE, Lim KH. Discrepancies between parental reports and clinical diagnoses of strabismus in Korean children. J AAPOS. 2012; 16(6):511-514.

55. He J, Lu L, Zou H, He X, Li Q, Wang W, Zhu J. Prevalence and causes of visual impairment and rate of wearing spectacles in schools for children of migrant workers in Shanghai, China. BMC Public Health. 2014; 14:1312.
56. Hegmann JP, Mash AJ, Spivey BE. Genetic analysis of human visual parameters in populations with varying incidences of strabismus. Am J Hum Genet. 1974; 26(5):549-562.
57. Hikmatullah BVS. Factors responsible for delayed presentation of strabismus in patients aging up to 16 years. Ophthalmol Update. 2018; 16(4):835-837.
58. Holland G. [On the time of onset and the cause of strabismus in early childhood]. Klin Monbl Augenheilkd. 1965; 147(4):498-508.
59. Horta-Santini JM, Vergara C, Colón-Casasnovas JE, Izquierdo NJ. Strabismus surgery at the Puerto Rico Medical Center: A brief report. Puerto Rico Health Sci J. 2011; 30(4):203-205.
60. Idrees Z, Dooley I, Fahy G. Horizontal strabismus surgical outcomes in a teaching hospital. Ir Med J. 2014; 107(6):176-178.
61. Iqbal S, Shafiq M, Zeeshan M, Nadeem HA, Jamshed M. Type of horizontal deviation in consanguinity. Pak J Ophthalmol 2018; 34(2):103-106.
62. Jiao Y, Zhu Y, Zhou Z, Jie Y, Wang J, Lu W, Wu X, Kan M, Zhao G. Strabismus surgery distribution during 10-year period in a tertiary hospital. Chin Med J (Engl). 2014; 127(16):2911-2914.
63. Jie Y, Xu Z, He Y, Wang N, Wang J, Lu W, Wu X, Jiao Y. A 4 year retrospective survey of strabismus surgery in Tongren Eye Centre Beijing. Ophthalmic Physiol Opt. 2010; 30(3):310-314.
64. Jonkers GH. Statistics on deviations of binocular imbalance. Ophthalmologica. 1960; 140:180-192.
65. Junejo SA, Ansari MA. Outcome of monocular surgery for horizontal strabismus in Hyderabad. Clin Ophthalmol. 2010; 4:269-273.
66. Junejo AY, Hassan M ul. Strabismus and its Types in Children of Age 6 to 15 Years Presenting at a Public Sector Hospital of Karachi. J Dow Univ Health Sci 2019; *13*:24-29.
67. Kac MJ, de Freitas Junior MB, Kac SI, de Andrade EP. [Frequency of ocular deviations at the strabismus sector of the Hospital do Servidor Publico Estadual de Sao Paulo.] Arq Beas Oftalmol. 2007; 70(6):939-942.
68. Kampanartsanyakorn S, Surachatkumtonekul T, Dulayajinda D, Jumroendararasmee M, Tongsae S. The outcomes of horizontal strabismus surgery and influencing factors of the surgical success. J Med Assoc Thai. 2005; 88 Suppl 9:S94-99.
69. Khorrami-Nejad M, Akbari MR, Khosravi B. The prevalence of strabismus types in strabismic Iranian patients. Clin Optometr. 2018; 10:19-24.
70. Kikudi Z, Maertens K, Kayembe L. [Strabismus and heterophoria: the situation in Zaire]. J Fr Ophtalmol. 1988; 11(11):765-768.
71. Kim IG, Park JM, Lee SJ. Factors associated with the direction of ocular deviation in sensory horizontal strabismus and unilateral organic ocular problems. Korean J Ophthalmol. 2012; 26(3):199-202.
72. Krzystkowa K, Pajakowa J. The sensorial state in divergent strabismus. In: Orthopt Proc 2nd Int Orthopt Congr, pp. 72-76, Ed: Mein J., Excerpta Medica, Amsterdam, 1971.
73. Lance P, Mitchell R. Australian contribution to international orthoptic association survey. Aust Orthopt J. 1983; 20:59-63.
74. Lee SH, Jung SJ, Ohn YH, Chang JH. Association between refractive errors and horizontal strabismus: the Korea National Health and Nutrition Examination Survey. J AAPOS. 2021; 25(6):340.e1-340.e7.
75. Li JH, Xie WF, Tian JN, Zhang LJ, Cao MM, Wang L. Changing strabismus surgery distribution at Shanxi Province Eye Hospital in Central China. J Pediatr Ophthalmol Strabismus. 2017; 54(2):112-116.

76. Louwagie CR, Diehl NN, Greenberg AE, Mohney BG. Is the incidence of infantile esotropia declining? A population-based study from Olmsted County, Minnesota, 1965 to 1994. Arch Ophthalmol. 2009; 127(2):200-203.
77. Magramm I, Schlossman A. Strabismus in patients over the age of 60 years. J Pediatr Ophthalmol Strabismus. 1991; 28(1):28-31.
78. Mahajan S, Gupta SK. Clinical study of concomitant strabismus. Perspect Med Res. 2020; 8(3):44-48.
79. Mahdi Z, Munami S, Shaikh ZA, Awan H, Wahab S. Pattern of Eye Diseases in Children at Secondary Level Eye Department in Karachi Pak J Ophthalmol. 2005; 22(3):145-151.
80. Martinez-Thompson JM, Diehl NN, Holmes JM, Mohney BG. Incidence, types, and lifetime risk of adult-onset strabismus. Ophthalmology. 2014; 121(4):877-882.
81. McNeil NL. Patterns on visual defects in children. Brit J Ophthalmol. 1955; 39(11):688-701.
82. Medghalchi A. [A study on prevalence of horizontal strabismus in patients under 14 years.] J Guilan Univ Med Sci. 2003; 12(47):80-85.
83. Merino P, Mateos C, Gómez De Liaño P, Franco G, Nieva I, Barreto A. [Horizontal sensory strabismus: characteristics and treatment results]. Arch Soc Esp Oftalmol. 2011; 86(11):358-362.
84. Merino Sanz P, Donoso Torres HE, Gómez de Liaño Sánchez P, Casco Guijarro J. Current trends of strabismus surgery in a tertiary hospital. Arch Soc Esp Oftalmol (Engl Ed). 2020; 95(5):217-222.
85. Meyer FA, Campusano C, Reyes J, Valdenegro JP. [Epidemiologic aspects of strabismus in Valparaíso, Chile]. Rev Med Chil. 1978; 106(9):718-720.
86. Moguel-Ancheita S, Ramírez-Sibaja S, Reyes-Pantoja SA, Orozco-Gómez LP. [Funciones visuomotoras e inteligencia posterior al tratamiento del estrabismo. Segunda fase.] Cir Cir. 2010; 78(6):470-475.
87. Monteiro S, Casal I, Vale C, Borges T, Miranda V, Parreira R, Meneres P. [Estrabismo em idade ambliogénica: estudo retrospetivo de 12 meses consecutivos de referenciação oftalmológica hospitalar.] Oftalmologia. 2016; 40(4):317-323.
88. Morales OY, Ghoul S, Muguercia GY, Delfino LRJ, Divasto CG. [Surgical behavior in the Ophthalmological Hospital “Friendship Algeria-Cuba.”] Rev Inf Cient. 2018; 97(1):10-18.
89. Mujica A, Navarro F. [Estudio estadistico de las affecciones de los ojos.] Rev Med Chile. 1897; 25(4):77-170.
90. Musa KO, Ikuomenisan SJ, Idowu OO, Salami MO, Olowoyeye AO. Spectrum of childhood strabismus seen at Lagos University Teaching Hospital, Lagos, Nigeria. Nig Qt J Hosp Med. 2017; 27(2):726-732.
91. Mustafa MSE. Common eye disorders in children attending Khartoum Eye Teaching Hospital. Thesis, 2006, University of Khartoum.
92. Nusz KJ, Mohney BG, Diehl NN. Female predominance in intermittent exotropia. Am J Ophthalmol. 2005; 140(3):546-547.
93. Mvilongo C, Omgbwa A, Nkidiaka C, Elom A, Hoffman W, Ebana C. Strabismus amblyopia in young Cameroonian at their first visit at Yaounde Hospital Centre-Essos. J Clin Exp Ophthalmol. 2016; 7:613.
94. Mvogo CE, Bella-Hiag AL, Ellong A, Mbarga BM, Epesse M. [Exotropia in black Cameroonians]. Sante. 1999; 9(5):289-292.
95. Najafi A. [Prevalence of different types of strabismus in Labbafinejad Hospital.] Med Sci. (J Islam Azad Univ.) 2007; 17(1):33-36.
96. Njambi L, Rita O, Kazim D, Sonia V**.** Prevalence and pattern of manifest strabismus in paediatric patients at CCBRT, Dar es Salaam, Tanzania**.** J Ophthalmol East Cent South Afri. (JOECSA) 2017; 21:9-12.

97. Nordlöw W. Age distribution of the onset of esotropia. Br J Ophthalmol. 1953; 37(10):593-600.
98. Nwachukwu H, Adio AO, Nathaniel GI, Musa KO. Pattern of Manifest Strabismus in Children Seen in a Tertiary Hospital in Rivers State, Nigeria. Int J Ophthalmol & Vis Sci. 2021; 6(4):209-214.
99. Okeigbemen VW, Momoh N. Ocular comorbidities in children with strabismus in Benin City. Sahel Med J. 2019; 22:13-17.
100. Oliveira BFTd, Bigolin S, BarretoSouza M, Polati M. [Sensorial strabismus: a study of 191 cases.] Arq Bras Oftalmol. 2006; 69(1):71-74.
101. Onyango J, Ntizahuvye S. Prevalence of strabismus and the outcomes of its management among children attending Ruharo Eye Center, South Western Uganda. J Ophthalmol East Cent South Afri. (JOECSA) 2017; 21:13-15.
102. Oummad H. [Le strabisme de l'adulte.] Thesis de Medecine. Univ. Mohammed V, Faculte de Medecine et de Pharmacie, Rabat, Morocco, 2011.
103. Pandey S, Pandey A, Bajracharya K, Thapa HB, Shrestha S, Bhari AM, Rai SKC. An assessment of surgical outcome with the influencing factors of horizontal strabismus surgery. Asian J Med Sci. 2017; 8(5)54-57.
104. Pickwell LD. The increase in convergence inadequacy with age. Ophthalmic Physiol Opt. 1985; 5(3):347-348.
105. Pineles SL, Repka MX, Velez FG, Yu F, Perez C, Sim D, Coleman AL. Prevalence of pediatric eye disease in the optumlabs data warehouse. Ophthalmic Epidemiol. 2022; 29(5):537-544.
106. Power B, Murphy M, Stokes J. The impact of strabismus surgery on Irish adults. Br Ir Orthopt J. 2018; 14(1):6-10.
107. Putri P, Julita. Profil Strabismus Horizontal di RSUP Dr. M Djamil Padang Januari – Desember 2017. Jurnal Kesehatan Andalas. 2020; 9(1):83-87.
108. Qanat AS, Alsuheili A, Alzahrani AM, Faydhi AA, Albadri A, Alhibshi N. Assessment of Different Types of Strabismus Among Pediatric Patients in a Tertiary Hospital in Jeddah. Cureus. 2020; 12(12):e11978.
109. Quere MA, Mehel E. 1995. [Epidemiologie. In: Les Strabismes de L'Adolescent et de L'Adulte.] V. 3.1. Cahiers de Sensorio-Motricite XXe Colloque, 1995. pp 3-7.
110. Rahmania N, van Rompay T, Morfeq H, Promelle V, Milazzo S. Surgical treatment of pediatric strabismus (PS): series of 148 patients. Acta Ophthalmol. 2016; 94:S256.
111. Rajavi Z, Sabbaghi H, Baghini AS, Yaseri M, Moein H, Akbarian S, Behradfar N, Hosseini S, Rabei HM, Sheibani K. Prevalence of amblyopia and refractive errors among primary school children. J Ophthalmic Vis Res. 2015; 10(4):408-416.
112. Rao GN, Sabnam S, Pal S, Rizwan H, Thakur B, Pal A. Prevalence of ocular morbidity among children aged 17 years or younger in the eastern India. Clin Ophthalmol. 2018; 12:1645-1652.
113. Razmjoo H, Haj-Yahya Y, Javic E, Abtahi SM, Mehrabi-Koushki A. Study of 100 children with strabismus admitted to Feyz hospital, Isfahan, Iran, in 2012-2013. J Isfahan Med School. 2015; 32:313.
114. Repka MX, Yu F, Coleman A. Strabismus among aged fee-for-service Medicare beneficiaries. J AAPOS. 2012; 16(6):495-500.
115. Repka MX, Lum F, Burugapalli B. Strabismus, strabismus surgery, and reoperation rate in the United States: Analysis from the IRIS registry. Ophthalmology. 2018; 125(10):1646-1653.
116. Reza BM, Reza MM, Mojgan K, Hassan LM. Pediatric strabismus: prevalence and surgical outcomes in Yazd, Iran. Guoji Yanke Zazhi (Int Eye Sci.) 2013; 13(8):1521-1524.
117. Ribeiro GdeB, Bach AG, Faria CM, Anastásia S, Almeida HC. Quality of life of patients with strabismus. Arq Bras Oftalmol. 2014; 77(2):110-113.

118. Rocha MNAM, Sanches A, Pessoa FF, Braz GS, Rego LP, Auad LJ, Ribeiro PCA. Clinical forms and risk factors associated with strabismus in visual binocularity. Rev Bras Oftalmol. 2016; 75(1):34-39.
119. Rohr JTD, Isaac CR, Correia CdS. Epidemiology of strabismus surgery in a public hospital of the Brazilian Federal District. Rev Bras Oftalmol. 2017; 76(5):250-254.
120. Sah SP, Sharma IP, Chaudhry M, Saikia M. Health-Related Quality of Life (HRQoL) in Young Adults with Strabismus in India. J Clin Diagn Res. 2017; 11(2):NC01-NC04.
121. Sarosh R, Khan A, Rashid O, Hakak B, un Nisa A, Sarosh P. Profile of strabismus at a tertiary care hospital in Kashmir. Int J Contemp Med Res. 2018; 5(6):F4-F7.
122. Schlossman A, Priestley BS. Role of heredity in etiology and treatment of strabismus. AMA Arch Ophthalmol. 1952; 47(1):1-20.
123. Sethee SK, FitzGerald DE, Krumholtz I. Exotropia in a pediatric population less than six years of age. J Behav Optometr. 2003; 14(6):149-157.
124. Shah MA, Khan S, Mohammad S. Presentation of childhood squint. J Postgrad Med Inst. 2002; 16:206-210.
125. Shah A, Sethi S, Ilyas O, Shah Z. The frequency of amblyopia & results of squint surgery in patients admitted in Khyber Teaching Hospital, Peshawar. Ophthalmology Update 2013; 11:109-111.
126. Siddiqui SN, Asif M, Iqbal S, Shah MA, Habiba U, Rabia M. Frequency of Different Types of Manifest Strabismus in Children. Al-Shifa J Ophthalmol. 2017; 13(2):86-92.
127. Sternberg A, Feher M. Five years experience with divergent squint. Ophthalmologica. 1958; 136(1):1-12.
128. Tinley C, Grötte R. Comitant horizontal strabismus in South African black and mixed race children--a clinic-based study. Ophthalmic Epidemiol. 2012; 19(2):89-94.
129. Tubing K, Patton T, Usharani L, Basar G, Tsapoe W, Lalhriatpuii ST, Satyabama M. Study of concomitant strabismus amongst the ethnic population of Manipur. IOSR J Dent Med Sci. 2014; 13(1):23-28.
130. van der Hoeve J. [Das Vorkommen von Strabismus an der Poliklinik für Augenheilkunde im “Ryks-Ziekenhuis zu Leiden” von Juli 1896 bis Dezember 1901.] Arch Augenh. 1902; 46:207-231.
131. Vázquez-Aguirre N, Arroyo-Yllanes ME, Fonte-Vázquez A. Dissociated deviation in sensorial strabismus. Rev Mex Oftalmol. 2018; 92(1):6-11.
132. Vlachou C, Mellou K, Tsaras K, Sparos L. [Determinants of strabismus frequency]. Arch Hellenic Med. 2003; 20(3):276-280.
133. Waardenburg PJ. Squint and heredity. Doc Ophthalmol. 1954; 7-8:422-494.
134. Waheeda-Azwa H, Norihan I, Tai ELM, Kueh YC, Shatriah I. Visual outcome and factors influencing surgical outcome of horizontal strabismus surgery in a teaching hospital in Malaysia: A 5-year experience. Taiwan J Ophthalmol. 2020; 10(4):278-283.
135. Wan X, Wan L, Jiang M, Ding Y, Wang Y, Zhang J. A retrospective survey of strabismus surgery in a tertiary eye center in northern China, 2014-2019. BMC Ophthalmol. 2021; 21(1):40.
136. Yang M, Chen J, Shen T, Kang Y, Deng D, Lin X, Wu H, Chen Q, Ye X, Li J, Yan J. Clinical Characteristics and Surgical Outcomes in Patients with Intermittent Exotropia: A Large Sample Study in South China. Medicine (Baltimore). 2016; 95(5):e2590.
137. Yin L, Chen X. [The causative diseases, common comorbidities and surgical procedures of 948 cases of horizontal sensory strabismus]. Zhonghua Yan Ke Za Zhi. 2018; 54(4):283-287.
138. Yu CB, Fan DS, Wong VW, Wong CY, Lam DS. Changing patterns of strabismus: a decade of experience in Hong Kong. Brit J Ophthalmol. 2002; 86(8):854-856.
139. Yu X, Ji Z, Yu H, Xu M, Xu J. Exotropia is the main pattern of childhood strabismus surgery in the South of China: A six-year clinical review. J Ophthalmol. 2016; 2016:1489537.

140. Zaki AAH, Keeney AH. The bony orbital walls in horizontal strabismus. Arch Ophthalmol-Chic. 1957; 57(3):418-424.
141. Zimmermann-Paiz MA, Ordonez-Rivas AM. [Frequency of various strabismus types in an ophthalmology facility of Guatemala City.] Rev Mex Oftalmol. 2013; 87(4):195-199.
